# Brachytherapy facilitates closure of malignant vesicovaginal fistulas: A case report and literature review

**DOI:** 10.1097/MD.0000000000044708

**Published:** 2025-11-14

**Authors:** Wentian Lyu, Shiqi Hu, Jianbo Li

**Affiliations:** a Department of Interventional Oncology, Wuhan Tongji Aerospace City Hospital, Wuhan, China.

**Keywords:** brachytherapy, cervical cancer, closure, malignant, vesicovaginal fistula

## Abstract

**Rationale:**

Malignant vesicovaginal fistula (MVF) usually result from the progression of bladder cancer or cervical cancer. Since the fistula openings are filled with malignant tumors, there are currently no effective treatment options or valuable research findings to achieve fistula healing, making it a difficult and intractable issue in clinical practice. In this report, we present an innovative and practical procedure to address this issue.

**Patient concerns:**

A patient with recurrent cervical cancer after treatment presented with persistent vaginal bleeding. On physical examination, the vagina was found to be filled with tumors that bled on palpation, and a large, hard mass was palpable in the abdomen.

**Diagnoses:**

The initial magnetic resonance imaging revealed that the patient’s lower abdomen and pelvic cavity were filled with tumors, the bladder was invaded by the tumor, and a vaginal fistula was present.

**Interventions:**

Sequential chemotherapy in combination with immunotherapy was carried out to facilitate the tumor downgrading, allowing subsequent interstitial brachytherapy conducted.

**Outcomes:**

Following a dose delivery of 24 to 40 Gy in 4 fractions over 2 weeks, the MVF was found unexpectively completely closed, and the hallmark symptom of urine leakage from the vargina resolved.

**Lessons:**

This case highlights the potential of brachytherapy as a promising and effective treatment modality for MVF. Further studies are warranted to validate these findings and establish standardized protocols.

## 1. Introduction

Malignant vesicovaginal fistula (MVF) is a severe complication arising from locally advanced bladder or cervical carcinoma, as defined by the AJCC Guidelines.^[1,2]^ MVF may remain asymptomatic when obstructed by tumor masses but can become symptomatic upon mass shrinkage induced by radiotherapy or chemotherapy, manifesting as urinary discharge from the vagina. The management of MVF typically involves specialized surgical or radiation oncology procedures, often pursued with palliative intent due to the malignant nature of the underlying disease, rather than with the goal of achieving fistula closure.^[3–5]^ To our knowledge, there is currently no evidence-based approach specifically aimed at achieving MVF closure. Furthermore, MVF is frequently misattributed to radiotherapy, despite the fact that radiotherapy plays critical roles in targeting cancer cells while sparing normal tissues.^[6–8]^ In this report, we present a case of cervical cancer – induced MVF that was treated using a sequential combination of medical oncology interventions and brachytherapy. This case demonstrates that brachytherapy may serve as a novel and effective therapeutic strategy for MVF. A novel hypothesis was further proposed, shedding light on the potential mechanisms and benefits of brachytherapy in the treatment of MVF.

## 2. Case description

### 2.1. Patient’s background

**Figure 1.**
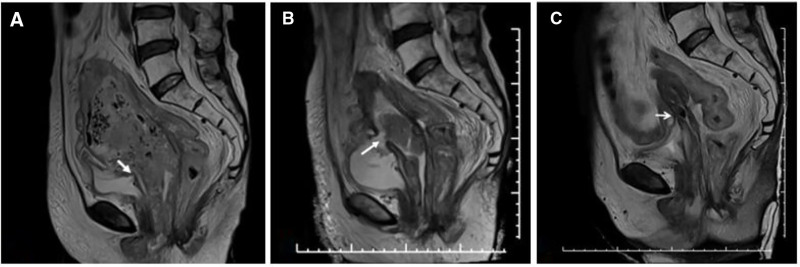
MRI of carcinoma invading reproductive organs. (A) Uterus, cervix and vargin were no longer visible. Bladder was partially invaded. White arrow indicated the lesion-blocked MVF; (B) after 4 cycle systematic treatment, tumor partially shrank. White arrow indicated the MVF was partially blocked, with signs of urine discharging from the vagina; (C) after 6 cycle systematic treatment, the tumor shrank significantly. White arrow indicated the MVF was no longer obstructed. MRI = magnetic resonance imaging, MVF = malignant vesicovaginal fistula.

In July 2022, an elderly patient was diagnosed with stage III cervical cancer. She underwent a treatment regimen consisting of 2 cycles of chemotherapy combined with immunotherapy, alongside external beam radiation therapy (EBRT) delivering a total dose of 4500 cGy in 25 fractions over a period of 5 weeks. Following her discharge from the hospital, she remained at home for 17 months, during which her condition deteriorated due to a relapse of the carcinoma. This relapse was accompanied by worsening symptoms, including vaginal bleeding, abdominal pain, the presence of an abdominal mass, and significant weight loss. In January 2024, the patient was readmitted to the hospital. Magnetic resonance imaging (MRI) scans indicated that the cancerous lesions had extensively invaded the lower abdomen, the entire reproductive system, the pelvic wall, and the bladder. Furthermore, the MRI revealed that all reproductive organs, including the uterus, cervix, and vagina, were no longer discernible (Fig. 1A). A gynecological examination confirmed the presence of hard lesions within the bleeding vaginal cavity and identified a large, firm mass in the lower abdomen. The patient was subsequently diagnosed with stage IV cervical cancer, presenting with vaginal bleeding, anemia, MVF, hypoproteinemia, and cancer-related pain. Her condition was assessed using the ECOG performance status scale as III grade.

### 2.2. Methods

Wuhan Tongji Aerospace Hospital Ethics Committee approved the study. Written informed consent was secured from the patient. All data generated or analyzed during this study are included in this published article.

In addition to best supportive care, a comprehensive systematic treatment strategy was proposed following consultation with a multidisciplinary tumor board. This strategy integrated medical oncology approaches, including chemotherapy and immunotherapy, combined with radiotherapy. In alignment with the NCCN guidelines,^[9,10]^ a chemotherapy regimen consisting of 6 cycles of paclitaxel and cisplatin was determined to be the most appropriate course of action. During the systematic treatment, the MVF, which had been obstructed by tumor lesions, remained asymptomatic until the fourth cycle of treatment that regression of the lesions revealed the presence of the MVF, manifesting as urine discharge from the vagina (Fig. 1B). Upon completion of the 6-cycle medical oncology treatment, significant tumor shrinkage was observed (Fig. 1C), accompanied by the resolution of hematological toxicity, allowing the followed brachytherapy initiated with the intent of enhancing local tumor control.

**Figure 2.**
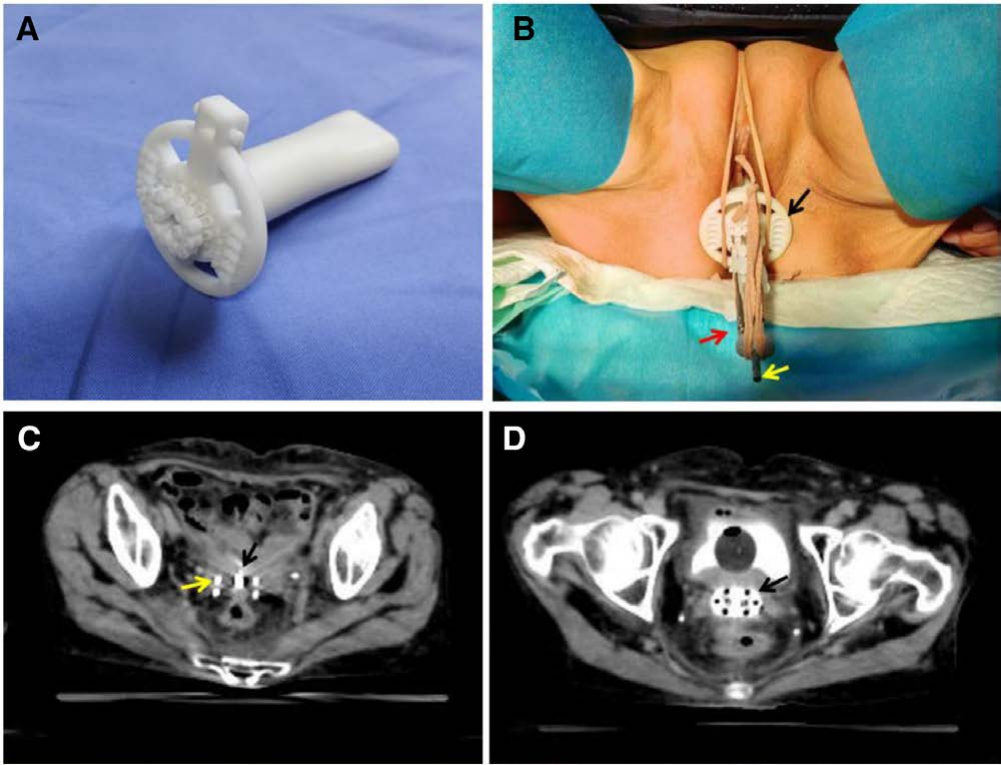
Brachytherapy procedure. (A) The interplantation template featuring multiple ducts designed for loading tandem components. (B) The picture of interplantation setup. The plastic template is loaded with tandem components, including interstitial needles, a vaginal colpostat and an intrauterine tandem. Black arrow points to the plastic template. Yellow arrow indicates the intrauterine tandem. Red arrow highlights the interstitial needle. (C) CT simulation image of interplantation procedure. Red arrow marks the interstitial needle. Black arrow indicates the intrauterine tandem. (D) CT simulation image of interplantation procedure. Black arrow indicates the vaginal colpostat. CT = computed tomography.

The high-dose-rate brachytherapy system (Elekta), loaded with the ^192^Ir radioactive isotope, was utilized for this treatment. A custom-designed plastic template with multiple ducts was fabricated to house tandem components, including applicators and interstitial needles, for precise radiation delivery. Each brachytherapy interplantation procedure was performed under the guidance of CT simulation (Fig. 2). The brachytherapy and dosing prescription regimen was carried out through a 3-dimensional treatment planning system, enabling both volumetric delineation of the target and dose calculation to be processed through the treatment planning system. Additionally, MRI immediately preceding each brachytherapy was performed in delineating residual tumor geometry and accessing therapeutic effect. The brachytherapy targeted the regions encompassing MVF, located 10 mm away from the implanted component. The prescribed dose was 30 Gy delivered in 5 fractions over 2 weeks, equivalent to a biologically effective dose of 40 Gy in 5 fractions at 2 Gy per fraction, as calculated using the linear-quadratic model. Meanwhile, the surface of the implanted component received a total dose of 50 Gy in 5 fractions.

### 2.3. Treatment outcome

**Figure 3.**
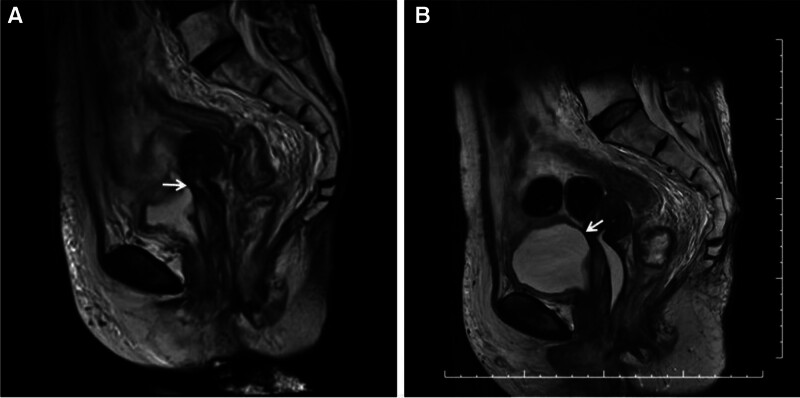
MVF MRI after fourth brachytherapy treatment. (A) MVF closure observed at the completion of brachytherapy, White arrow indicated the site of MVF; (B) MVF closure observed 1 month after the fourth brachytherapy treatment. White arrow indicated the site of MVF. MRI = magnetic resonance imaging, MVF = malignant vesicovaginal fistula.

In this case, the rectum was free from tumor involvement, and was carefully spared during the brachytherapy procedure to adhere to the organ-at-risk constraint the organ-at-risk constraint, with the 2-cc rectal dose maintained at ≤65 to 75 Gy. Unexpectedly, following the fourth treatment procedure, urine was observed to flow out of the urethra canal instead of the vargin. MRI immediately revealed the closure of the MVF, which was consistent with the observed urinary discharge (Fig. 3). Concurrently, the patient reported labial pain, likely attributed to radiation-induced tissue vulnerability. This complication ultimately precluded the administration of the fifth brachytherapy procedure.

After brachytherapy, the patient proceeded to receive EBRT, with the target volumes encompassing the entire uterus, vagina, bladder, and lymphatic drainage regions. This approach was intended to control residual tumor cells, delay tumor metastasis and recurrence, and extend overall survival. Upon completion of EBRT, tumor response was evaluated using imaging studies, colposcopy, and hematologic tests. In accordance with the RECIST criteria, the treatment response was assessed as complete response, indicating the complete disappearance of all tumors. The patient subsequently entered the follow-up phase.

### 2.4. Follow-up

Following treatment completion, the patient underwent regular follow-up evaluations every 3 months, with abdominal MRI scans obtained at each visit. During the follow-up period, no signs of vaginal urinary leakage, recurrence of vaginal or uterine cancer, or cystitis were observed. MRI confirmed healing of the MVF. Nine months after treatment conclusion, the patient developed metastases to multiple organs, including the peritoneal cavity, lungs, and brain. The patient declined further therapeutic intervention and expired 3 months after the detection of these metastases.

## 3. Discussion

MVF is challenging to treat surgically due to its aggressive behavior. Of note, there is limited literature demonstrating the treatment of MVF using brachytherapy. This report outlines a successful treatment protocol for MVF resulting from cervical cancer, with brachytherapy playing a pivotal role in the healing of MVF. Following 6 cycles of systemic therapy involving chemotherapy combined with immunotherapy, MRI imaging revealed significant shrinkage of the lesions in both the cervix and the MVF. This improvement allowed for the subsequent application of radiation therapy to achieve an optimal outcome. Radiation treatment includes brachytherapy and EBRT.^[11]^ EBRT is directed at regions containing the gross tumor and the lymphatic drainage area, administered with either prophylactic irradiation intention or radical aim depending on individual consideration. In this case, the EBRT regimen theoretically involved delivering radiation to the lower abdomen and reproductive system. However, the conventional dose schedule was unsuitable due to its inability to meet tissue constraints. Furthermore, the quality control and assurance in EBRT required precise management of bladder and rectum filling to minimize the overexposure of the bowel. In this case, EBRT is not appropriate to deliver an adequate radiation dose to lesion-surrounded vesicovaginal fistula, as the bladder could not be adequately filled and spared during radiation.

To effectively and safely treat the remaining lesions, a procedure capable of sparing normal tissues was required. Brachytherapy emerged as an appropriate approach to meet this need.^[12,13]^ Besides delivering high-dose radiation to the target lesions, brachytherapy is characterized by a steep dose decline, unlike EBRT, brachytherapy does not require stringent management of the bladder and rectum, simplifying quality control. In this case, interstitial brachytherapy was employed to target the uterus, cervix, and vagina, including the site of the MVF. A dose regimen of 2400 cGy was administered in 4 fractions over 2 weeks. The closure of the MVF was monitored using both MRI and clinical evaluation, revealing an unexpected and meaningful outcome.

### 3.1. “Take-away” lessons

The hypothesized mechanism of brachytherapy’s effect on MVF, as presented in this work, can be summarized as follows:

1. Initial reduction in tumor mass: During the first phase of treatment, which includes systemic therapies such as chemotherapy and immunotherapy, a significant portion of the tumor mass within the sentinel system shrinks. This reduction in size consequently decreases the dimensions of the MVF.
2. Brachytherapy application: During the brachytherapy phase, either applicators or interplantation needles are positioned directly against the lesions. A dose schedule of 600 to 1000 cGy per fraction is administered, typically totaling 4 fractions.
3. Lesion destruction and healing: The brachytherapy regimen destroys the lesions while simultaneously inducing inflammation and edema. This process initiates the adhesion and closure of the MVF, ultimately leading to its healing.

### 3.2. Limitations

Unlike the MVF closure achieved in this study, which was based on treating shrunken lesions, fistulas filled with large lesions that invade surrounding tissues may be challenging to address with brachytherapy alone. Due to the nonuniform dose distribution characteristic of brachytherapy, which is concerned with the radiobiological dose-effect relationship rather than the delivery dose alone, as such, even the dose regimen was prescripted higher than the one used in this study might still fail to sufficiently reduce the lesions. The key to success lies in systematically minimizing the MVF through comprehensive treatment to create conditions suitable for effective brachytherapy.

Of note, the follow-up results in this study indicate that brachytherapy targeting the MVF promoted healing of the fistula while keeping surrounding organs within radiation constraints. However, it did not extend overall survival due to uncontrolled cancer metastasis. Therefore, a systematic therapeutic strategy integrated with individualized considerations remains crucial for prolonging overall survival.

### 3.3. Future prospects

Management of MVF while preserving urological and gynecological function remains an unmet clinical need, as this challenge often exceeds the capabilities of conventional surgical interventions. Building on the hypothesis proposed in this study regarding the potential role of brachytherapy in treating MVF, multiple areas require further exploration – including radiobiological mechanisms, radiophysical optimization, and clinical trial validation. These investigations aim to achieve precise radiation delivery to MVF lesions, thereby inducing tumor shrinkage, promoting fistula closure, and facilitating healing through integration with systemic therapies.

## 4. Conclusion

MVF, a refractory complication, significantly impacts not only disease control but also quality of life. To date, there is no high-grade, evidence-based approach to guide the treatment of MVF. However, brachytherapy, as a precise and high-dose delivery procedure, may offer a promising option, as demonstrated in this report. On one hand, brachytherapy can reduce the size of MVF by delivering lethal radiation to the target area while sparing surrounding normal tissues. On the other hand, the high-dose delivery of 2400 to 4000 cGy in 4 fractions can induce inflammation and edema, potentially promoting adhesion and closure of the fistula. For larger lesions involving MVF, personalized consideration including medical oncology and EBRT aiming to downgrade cancer should be taken into account firstly to create a more favorable condition for subsequent brachytherapy. The findings presented here suggest a potential mechanism for brachytherapy-induced MVF healing, highlighting the need for further research to explore the relationship between MVF treatment and brachytherapy.

## Acknowledgments

I would like to express my sincere appreciation to Shuihong Hu who gives permission to be named for his expertise and assistance in needle interplantation during the brachytherapy procedures.

## Author contributions

**Conceptualization:** Wentian Lyu.

**Formal analysis:** Shiqi Hu.

**Investigation:** Jianbo Li.

**Methodology:** Wentian Lyu.

**Resources:** Jianbo Li.

**Supervision:** Wentian Lyu.

**Writing – original draft:** Wentian Lyu.

**Writing – review & editing:** Wentian Lyu.
